# Emerging Putative Associations between Non-Coding RNAs and Protein-Coding Genes in Neuropathic Pain: Added Value from Reusing Microarray Data

**DOI:** 10.3389/fneur.2016.00168

**Published:** 2016-10-18

**Authors:** Hemalatha B. Raju, Nicholas F. Tsinoremas, Enrico Capobianco

**Affiliations:** ^1^Center for Computational Science, University of Miami Miller School of Medicine, Miami, FL, USA; ^2^Human Genetics and Genomic Graduate Program, University of Miami Miller School of Medicine, Miami, FL, USA; ^3^Department of Medicine, University of Miami Miller School of Medicine, Miami, FL, USA

**Keywords:** neuropathic pain, microarray data reuse, differential expression, time-course profiling, pathway analysis, non-coding RNAs, networks

## Abstract

Regeneration of injured nerves is likely occurring in the peripheral nervous system, but not in the central nervous system. Although protein-coding gene expression has been assessed during nerve regeneration, little is currently known about the role of non-coding RNAs (ncRNAs). This leaves open questions about the potential effects of ncRNAs at transcriptome level. Due to the limited availability of human neuropathic pain (NP) data, we have identified the most comprehensive time-course gene expression profile referred to sciatic nerve (SN) injury and studied in a rat model using two neuronal tissues, namely dorsal root ganglion (DRG) and SN. We have developed a methodology to identify differentially expressed bioentities starting from microarray probes and repurposing them to annotate ncRNAs, while analyzing the expression profiles of protein-coding genes. The approach is designed to reuse microarray data and perform first profiling and then meta-analysis through three main steps. First, we used contextual analysis to identify what we considered putative or potential protein-coding targets for selected ncRNAs. Relevance was therefore assigned to differential expression of neighbor protein-coding genes, with neighborhood defined by a fixed genomic distance from long or antisense ncRNA loci, and of parental genes associated with pseudogenes. Second, connectivity among putative targets was used to build networks, in turn useful to conduct inference at interactomic scale. Last, network paths were annotated to assess relevance to NP. We found significant differential expression in long-intergenic ncRNAs (32 lincRNAs in SN and 8 in DRG), antisense RNA (31 asRNA in SN and 12 in DRG), and pseudogenes (456 in SN and 56 in DRG). In particular, contextual analysis centered on pseudogenes revealed some targets with known association to neurodegeneration and/or neurogenesis processes. While modules of the olfactory receptors were clearly identified in protein–protein interaction networks, other connectivity paths were identified between proteins already investigated in studies on disorders, such as Parkinson, Down syndrome, Huntington disease, and Alzheimer. Our findings suggest the importance of reusing gene expression data by meta-analysis approaches.

## Introduction

Neuropathic pain (NP) is caused by intense damage brought to nervous system and has a differentiated origin (1). The cause could be either injury affecting the somatosensory nervous system or the damages to either the peripheral nervous system or the central nervous system (CNS) (2). NP is categorized into peripheral and central. The central NP is found implicated in spinal cord injury and in a few stroke cases. For instance, a significant presence of NP was detected in patients with spinal cord injury that experienced chronic pain (3). Peripheral nerve injuries cause damage to both the nerve and the adjacent connective tissue, but the injured nerves can regenerate in the peripheral nervous system by the activation of the intrinsic growth capacity of neurons (4). However, injured nerves generally fail to regenerate in the CNS (5). Various studies on sciatic nerve (SN) injury have been reported with reference to the rat model (6, 7), which is considered as the reference in our work too. Consisting of mixed populations of motor and sensory axons, SN is commonly addressed in nerve regeneration studies. The sensory neurons extending into SN are located in the L4–L6, lumbar vertebrae fourth through sixth dorsal root ganglion (DRG).

Due to the relevance of measuring the expression levels of both DRG and SN tissues, an opportunity is currently offered by many available microarray data, through which cross-reference studies and comparative evaluations can be performed. Meta-analyses have been conducted in many independent experiments to detect genes regulated by chronic pain states (8). With a focus limited to mRNA transcripts, little or no relevance was assigned to non-coding RNAs (ncRNAs) (9). A couple of exceptions were due to microRNA profiling based on SN, and to lncRNAs with temporal profile monitored after DRG injury, respectively (6, 9). This knowledge gap has motivated our work. An opportunity was offered by the reuse of data from open access resources, with the aim of overcoming the paucity of evidences due to limited experiments, high costs, and technical difficulties.

Peripheral inflammation and nerve injury cause changes in the expression of some miRNAs (7, 9, 10). Other ncRNAs might be considered relevant too, as they may regulate genomic expression to an extent yet largely unknown (11). Most studies have been based on deep sequencing and associated bioinformatic analyses lacking clear reference to functions, but reporting on target genes. Naturally enough, knockout studies may advance knowledge, but human transcripts are problematic to examine *in vivo* (12). Despite problems with cis-specific functions, mechanisms explaining regulatory functions have been shown especially with reference to epigenetic modulation, protein scaffolding, miRNA sequestration, competitive inhibition, etc. These processes include the ability to modulate target gene transcription (13).

The functional significance of such regulators has been discussed in physiology, development, and also various disease processes (14–16). However, up to now, relatively few ncRNAs have been studied. For instance, the expression of some antisense transcripts is linked to the activity of neighbor genes (17), but the regulation of gene expression by antisense transcripts is not acting as an isolated but rather as part of integrated mechanisms to achieve complex regulatory effects (18). Additional consideration deserves the complexity of lncRNAs in relation with the architecture of RNA-binding proteins, due to the presence of multiple RNA-binding domains that may recognize distinct “targets” (19). There is currently little evidence for direct interaction between lncRNAs and DNA. RNA–DNA hybrids or triplex structures can allow single strands of RNA to interact with DNA duplexes by base–pair interactions. These direct RNA–DNA interactions could efficiently and selectively target RNA signals to genomic loci (20).

Antisense biotypes were identified in the mammalian nervous system, including in chronic pain-related regions (21). This holds for the Kcna2 antisense RNA in DRG, for instance, which is overexpressed after peripheral nerve injury (22). In NP regulation, the relationships between protein-coding genes and ncRNA expression are also important for the critical role that the former play, that of target genes. Our study looks at such associations, focusing on the analysis of differential expression in both regulators and targets.

We recently reported work with preliminary analysis of the dataset GSE30165 (23). The rationale was to identify mRNA isoforms and ncRNAs underlying NP, using only the top 250 differentially expressed biotypes. In the present work, starting from ncRNAs classification and curation in relation to NP, we provided further analysis, i.e., contextual annotations for lncRNAs, suggesting a strategy to strengthen inference about the potential functional role of ncRNAs. In particular, since overexpression of ncRNAs acts toward reduction of the expression of potential targets, our evidences suggest to pay attention to putative targets potentially associated with regulatory and signaling NP mechanisms, also studied in relation with brain diseases.

## Materials and Methods

### Microarray Data

The raw microarray data were downloaded from the Gene Expression Omnibus database^1^ (GEO), platform Agilent-014879: Whole Rat Genome Microarray 4 × 44 K G4131F. Gene expression signatures for 30 samples were generated, including L4-6 DRG and proximal SN tissues (0.5 cm) at day 0, day 1, day 4, day 7, and day 14 after SN resection. This dataset included three samples in each tissue, DRG, and SN, and the expression levels were measured for all samples at five time points.

The differential expression was computed with GeneSpring (GX V 12.6). The time baseline was assigned the same way in both studies, with DRG and SN. Given the start day, 0 day = *b*, further measurements that were taken at successive intervals were redefined as: I1 = 1 day − *b*, I2 = 4 day − *b*, I3 = 7 day − *b*, I4 = 14 day − *b*. Sample groups thus appear according to time point in each tissue, for a total of five groups. Significance was assigned when differences in expression values between the groups had fold change (FC) >1.5, and *t*-test *p*-values <0.05, then adjusted for multiple testing by the false discovery rate (FDR) or Benjamini and Hochberg method (24). The probe sets and genes correspondence was established by FDR cutoff *q* < 0.05, and this is a standard way to bypass the problem of probes mapping to multiple genes. The outcome of this process (see also Table S8 in Supplementary Material) was the gene set called DEGs, retained for downstream analysis.

Of course, the stringency of these criteria influences the relative abundance or paucity of evidences that allow downstream analysis and thus inference; however, the goal of capturing biotypes other than genes, say ncRNA, suggests considering that relatively low expression values should be expected to characterize them.

### Pathway Analysis

Pathway analysis was obtained using the tool GeneGo Metacore™ (Thomson Reuters Corporation, New York, NY, USA). The DEGs identified by GeneSpring analysis were uploaded to the GeneGo website, and core analysis was applied to obtain the list of activated pathways, using 0.05 as a threshold for *p*-values. While we rely on established tools for protein-coding gene annotations, i.e., DEGs enriching for pathway terms, we need to work at a more empirical level for the ncRNAs, as explained below.

### NcRNA Annotation

The differentially expressed detections were annotated with both protein-coding and non-coding biotypes, as provided by Ensembl rel. 77 (25), Rattus_norvegicus.Rnor_6.0.79.gtf, and Mus_musculus.GRCm38.76.gtf. In the non-coding category, annotations regarding lncRNAs were provided for pseudogenes, lincRNAs, and asRNA – considered of major interest in our work. Due to the categories appearing under the label pseudogene, we aimed to have one single group, and thus the processed pseudogenes were consolidated into the final pseudogene group. Below, we explain the annotation strategy for these three major categories. Lastly, we indicate the use of targets to build protein–protein interaction networks (PINs).

#### LincRNA Neighbors

The contextual analysis of lincRNAs with respect to the DEG targets was established on the basis of the physical proximal distance on the chromosome. The mode of action of lncRNAs is generally classified into cis and trans regulation [see among other references, Ling et al. (26)]. This depends on whether the lncRNA regulates neighboring genes, i.e., genes on the same chromosomal regions where they are located, or instead distant genes, i.e., on other chromosomes. Notably, lncRNAs interact directly or indirectly with genomic DNA elements, often through proteins that perform specific biological functions, and also through other neighbor lncRNAs in a coordinated way. Note that lncRNAs may target proteins to exert their trans effects, but in such case to know what factors determine the RNA–protein interaction requires further study, here not pursued.

Locations of DE lincRNAs were obtained from Rattus_norvegicus.Rnor_6.0.79.gtf and Mus_musculus.GRCm38.76.gtf, i.e., gene transfer format files downloaded from Ensembl release 77. Two text files, one with lincRNA genomic locations (start and end positions) and another with protein-coding genomic locations (start and end positions), allowed lincRNA neighbors to be annotated. Genes at both left and right sides of starting and ending positions of lincRNAs, and within ±3 Mbps regions, were considered as putative targets. Note that this interval is arbitrary, and there is not a universally accepted definition of such range. In an attempt to explore neighbors of the lncRNA locus that confidently allow putative targets to be identified, we assigned priority to targets located at the closest possible distance from the locus of interest (see Results).

#### asRNA

They are among the most important categories of lncRNAs and known to regulate protein-coding genes on the opposite strand. Using information on the genomic coordinates of the genes and their coding potential obtained from the GTF files, we looked into the specific protein-coding genes that were on the opposite strand to DE asRNA in both tissues. Two text files were created for rat (i) asRNA file: a text file describing gene_id, chromosome, start, end, strand, gene_name obtained from Rattus_norvegicus.Rnor_6.0.79.gtf and (ii) GTF file: a text file that has all the genomic information but not restricted to asRNAs. These two text files were parsed through basic Perl scripts and for different steps to obtain the asRNA targets. Basically, this involved checking whether the chromosome numbers are the same in both asRNA and GTF text files and if the strands are opposite, then the ordering of the start position of genes in the asRNA file compared to the end genomic location, and also of the start position of genes in GTF file compared to the end genomic location. Finally, only the gene names from the GTF file on the opposite strand to the corresponding gene in the asRNA file were retained. Then, the same exact protocol was followed to obtain asRNA targets using the mouse reference, Mus_musculus.GRCm38.76.gtf.

#### Pseudogene–Parental Gene Annotations

The differentially expressed pseudogenes obtained for both tissues were annotated with respect to their parental protein-coding genes. The pseudogene sequences were mapped against the protein-coding gene sequences using BLAST (27), and only the unique hits were assigned to parental gene–pseudogene associations. Fasta sequences for protein-coding genes and pseudogenes were downloaded from the rat reference, Rattus_norvegicus.Rnor_6.0.cdna.all.fa. These two sets of sequences were mapped against each other using BLAST and the parameter “-max_target_seqs:1” to detect only those protein-coding genes that have a high-level of sequence homology to the pseudogenes used as query. Finally, the output obtained from BLAST was analyzed to overcome the multiple associations, i.e., when a pseudogene aligns to multiple protein-coding genes. The BLAST output was filtered using the “sort –u” option, which sorts the file and pipes the output that contains only unique hits. Therefore, only unique hits were considered as our final list for the downstream analysis. Similar steps were followed to extract parental genes from the mouse reference Mus_musculus.GRCm38.cdna.all.fa as well, as reported in parenthesis in Table 1. Further control of the selected parental genes aimed to identify DE in either neuronal tissue.

**Table 1:** **Classification of bioentities (source: Ensembl) – combined gene annotations for differentially expressed bioentities at different time points for the two tissues (SN, DRG)**.

Biotype	SN annotations				DRG annotations			
	Interval 1 SN_I1	Interval 2 SN_I2	Interval 3 SN_I3	Interval 4 SN_I4	Interval 1 DRG_I1	Interval 2 DRG_I2	Interval 3 DRG_I3	Interval 4 DRG_I4
Protein-coding gene	6392	5722	6436	3788	619	500	1274	2811
Pseudogene	366 **(172)**	303 **(157)**	291 **(135)**	188 **(92)**	19 **(9)**	10 **(6)**	34 **(15)**	98 **(55)**
lincRNA	31 **(22)**	27 **(21)**	30 **(22)**	14 **(12)**	1	1	3 **(2)**	10 **(6)**
Antisense	18 **(16)**	16 **(15)**	14 **(13)**	11 **(10)**	0	0	3 **(3)**	12 **(12)**

*Numbers in parentheses indicate ncRNAs from mouse reference, compared to rat evidences*.

*A pathway related to immune response that appears in SN_I1, SN_I3, SN_I4, DRG_I1, DRG_I2, and DRG_I3 involves chemokines, indicating a possible role in neuroinflammation and in alteration of neuronal plasticity (28)*.

### Target-Driven Protein–Protein Interaction Networks

The parental protein genes found differentially expressed in either of the neuronal tissues were used to generate the PIN using STRING^2^ (V. 9.1). This is a known state-of-the-art database reporting both experimental and predicted protein–protein interactions. This network was functional to our downstream contextual analysis, i.e., the contextualization of the identified targets with respect to biological processes and pathways.

### Rat Model and Mouse Reference

We used Mus_musculus.GRCm38.cdna.all.fa and Rattus_norvegicus.Rnor_6.0.cdna.all.fa from Ensembl (rel 77). The mouse was used as a reference only.

## Results

### Probe and Gene Annotation

The microarray dataset was profiled for the DEGs in both neuronal tissues – DRG and SN – at different time points (see Table 1). In addition, annotated differentially expressed probes and ncRNAs are uniquely obtained using the mouse reference (see Materials and Methods for details and bold numbers in parentheses reported in Table 1).

We detected 32 unique lincRNAs in SN and 8 unique lincRNAs in DRG. Then, we detected 31 unique antisense in SN and 12 unique antisense in DRG. Such antisense detections in SN and in DRG were not significantly associated with targets; hence, they were no longer explored. We additionally identified 452 unique pseudogenes in SN and 56 unique pseudogenes in DRG, subsequently investigated for their protein-coding targets.

### Pathways

**Figure 1:**
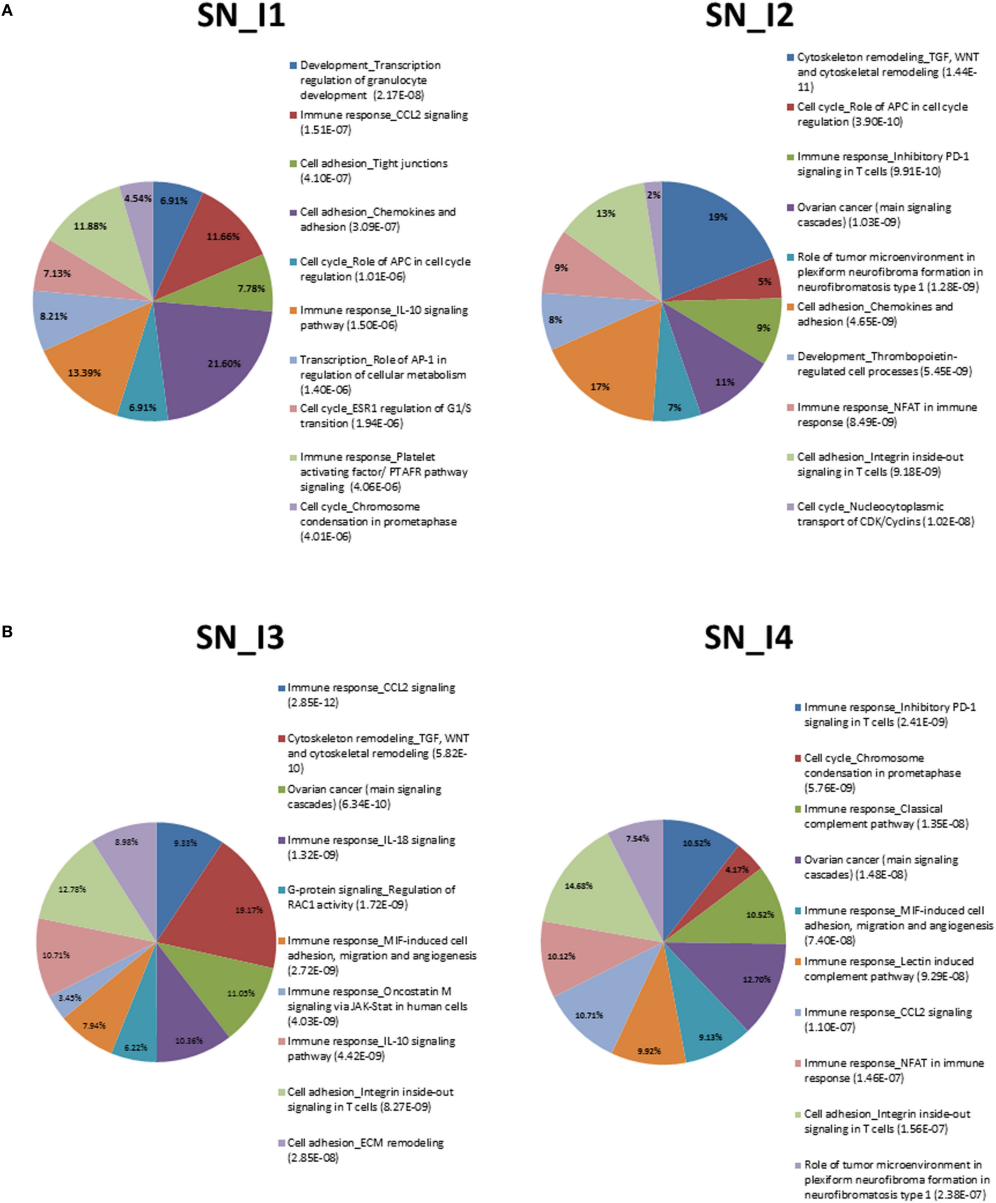
**(A)** Summary of top-10 pathways in SN_I1 (left) and SN_I2 (right). Percentage inside the pies represents enriched genes. FDR-corrected enrichment values are reported with listed pathway terms. **(B)** Summary of top-10 pathways in SN_I3 (left) and SN_I4 (right). Percentage inside the pies represents enriched genes. FDR-corrected enrichment values are reported with listed pathway terms.

**Figure 2:**
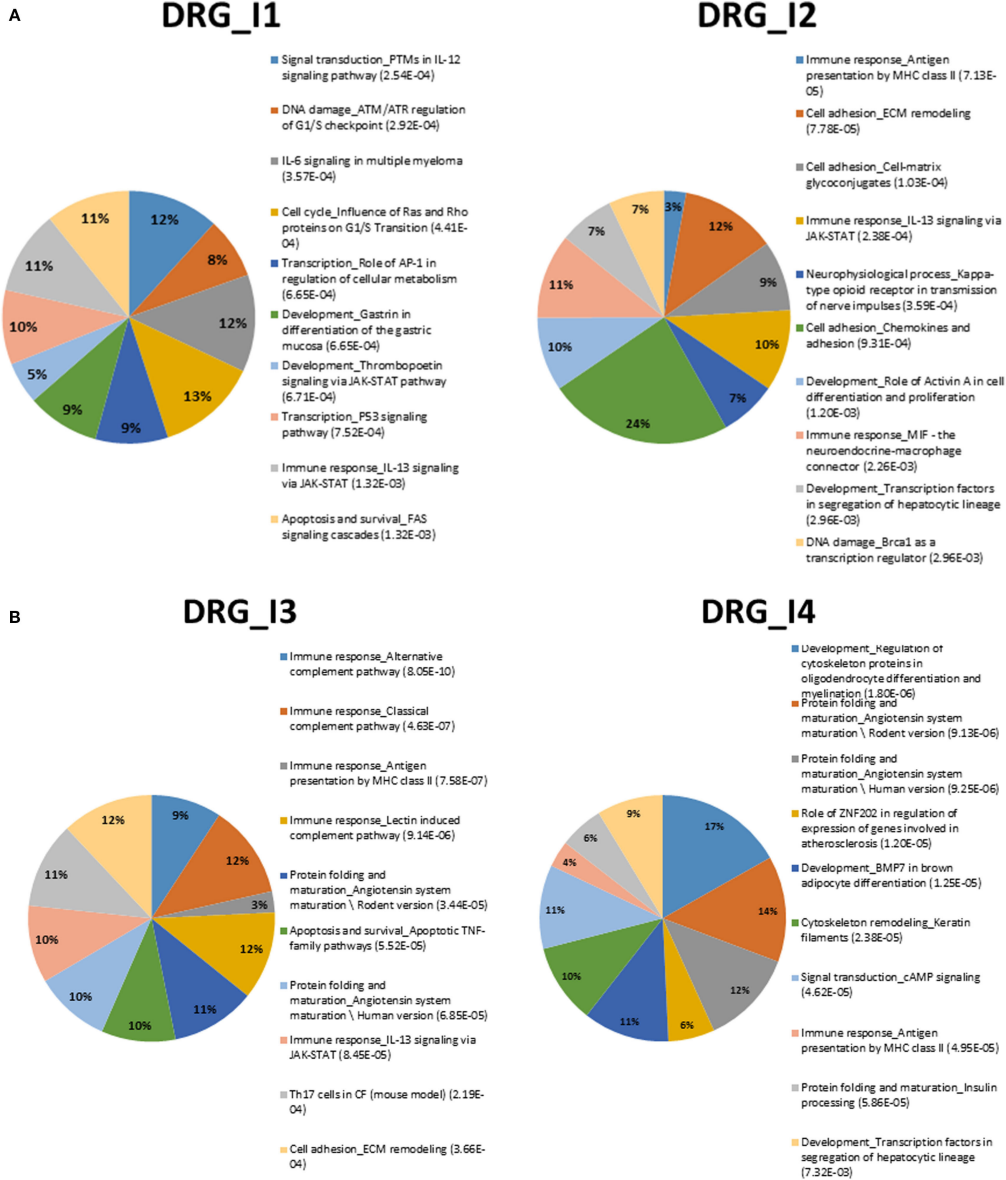
**(A)** Summary of top-10 pathways in DRG_I1 (left) and DRG_I2 (right). Percentage inside the pies represents enriched genes. FDR-corrected enrichment values are reported with listed pathway terms. **(B)** Summary of top-10 pathways in DRG_I3 (left) and DRG_I4 (right). Percentage inside the pies represents enriched genes. FDR-corrected enrichment values are reported with listed pathway terms.

Pathway analysis was conducted with the tool *GeneGo*, delivering evidences displayed in Figures 1A,B and 2A,B and summarized in the top-10 annotated terms for both tissues, DRG and SN. The enriching DEG sets appear in supplemental files (see Tables S6 and S7 in Supplementary Material for complete lists of annotated terms). Also, fractions of genes enriching pathway terms are reported inside the plots, while the listed terms are sorted by FDR-corrected enrichment values (from best, at the top).

We can observe among the terms in Figure 1A the one annotated as *neurophysiological process_Kappa-type opioid receptor in transmission of nerve impulses*, especially involved in DRG_I2. The relevance of this term is associated to CREM (activators), AP-1, and P/Q-type calcium channel alpha-1A subunit. The latter gene is a voltage-dependent calcium channel that mediates the entry of calcium ions into excitable cells and is involved in various calcium-dependent processes, like muscle contraction, neurotransmitter release, and gene expression. It is also primarily expressed in neuronal tissue, and it was shown that mutations to this gene could potentially cause two neurological disorders (29).

We also noted recurrence of *cell adhesion_Integrin inside-out signaling in T cells* at SN_I2, SN_I3, and SN_4, but not at SN_I1. This may indicate a possible role for the integrin-regulated cell adhesion in tissue morphogenesis and wound healing, together with the regulation of cell growth and differentiation, whereas the temporal pattern points to recurrence after injury.

Another interesting pathway is *immune_response_CCL2_signaling*, which is involved mainly in SN and refers to chemokines previously shown to be upregulated in injured DRG neurons under different NP models (30–33). We found enrichment of *immune response_NFAT* in SN_I2, SN_I4 and *signal transduction_cAMP signaling* in DRG_I4 by calcineurin A (catalytic), which is a calcium and calmodulin dependent serine/threonine protein phosphatase. This gene is relevant for CNS functions, such as neurite extension, synaptic plasticity, learning, and memory (34, 35).

Most pathways in SN show gene enrichment for ERK1/2, a key extracellular-signal-regulated protein kinase. The abnormal hyper-phosphorylation of tau, a gene that encodes the microtubule-associated protein tau, promotes microtubule stability and functions as a linker protein between axonal microtubules and neural plasma membrane components. Its mutation in Alzheimer’s disease (AD) has been shown to involve the ERK of the mitogen-activated protein (MAP) kinase family. Both the intracellular and regional distribution of the active forms of both MEK1/2 and ERK1/2, and the accumulation of p-MEK1/2 and p-ERK1/2 are known, the latter found in cases with stages I–III neurofibrillary degeneration (36).

### Contextual Analysis of lincRNAs and Detection of the Neighboring DEGs

We detected the differentially expressed ncRNAs across DRG and SN. Further classification was operated into three lncRNA categories, namely pseudogenes, lincRNAs, and asRNAs. LincRNA sequences categorized into different types on the basis of their genomic location with respect to protein-coding genes have been explored in very few studies (9, 22). The lincRNAs, detected by using both mouse and rat references, were manually curated to verify whether they could reveal potential candidate therapeutic targets for NP. In particular, potential association with the neighboring DEG targets was selectively investigated, i.e., within ±3 Mbps.

Table 2 shows the list of DEGs with respect to lincRNAs in both tissues and the corresponding annotations. The directional location of putative targets with respect to the genomic location of the lincRNAs is also reported. Analysis of lincRNAs and their neighboring DEGs revealed about 26 potential protein-coding targets in SN and 4 in DRG. In particular, we enabled a search for context-related information for each candidate bioentity, and after further manual curation of these 30 targets, we found examples, such as RGS22, RGS18 (regulators of G-protein signaling), and STIM2 (stromal interaction molecule 2), among the candidate DEGs in SN (indicated by *), with the potential to be targets of lincRNAs.

**Table 2:** **lincRNAs and gene targets in SN and DRG (Ensembl rel 77)**.

	lincRNA		Left		Right		
	Context-rich	Target	−1 MB	Exact location	1 MB	2 MB	3 MB
SN	RGD1562521	Rgs22^a^		274577	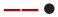		
SN	Rn50_X_0667.2	Rgs18^a^	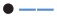	217956			
SN	Gm26825	Stim2^a^	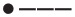	487168			
	**Annotated evidences**					
SN	Fam9b	Vps13b	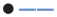	274577			
SN	Rn50_X_0711.1	Creb1		116889	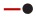		
SN	Ct55	Edem3	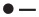	84281			
SN	Rn50_14_0846.1	Suco		242915	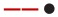		
SN	Rn50_13_0828.1	Zfyve28	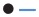	18891			
SN	Fam178b	Ptchd1		253992	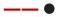		
SN	Rn50_13_0839.5	Pof1b		145307	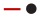		
SN	Rn50_7_1163.2	Gpd1		699513	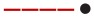		
SN	Ino80dos	Rb1cc1		609632	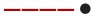		
SN	RP23-61N4.3	Gng11		602367	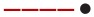		
SN	Gm26673	Thsd7a		862550	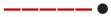		
SN	Gm26827	Peg3		413420	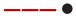		
SN	Gm20204	Arhgef7		493494	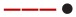		
SN	Gm28933	Rdh14		28368	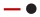		
SN	Gm26723	Rdh14		130201	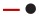		
SN	Gm26819	Klf6		1620120	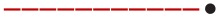		
SN	Yam1	Tfb1m		965615	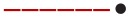		
SN	Gm26823	Cul2		303188	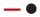		
SN	Gm4221	Zeb1		416081	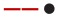		
SN	4731419I09Rik	Hey1		771424	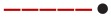		
SN	A530017D24Rik	Ccm2		139918	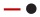		
SN	1700086L19Rik	Klhl29		2852122	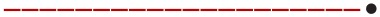		
SN	C130071C03Rik	Wdr37		85451	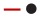		
DRG	1700020I14Rik	Itga8		354118	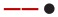		
DRG	Rn50_X_0744.2	Edem3	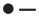	39848			
DRG	Rn50_13_0853.1	Chm		974768	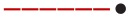		
DRG	Rn50_7_1164.1	Lhfpl1	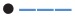	678582			

*DEG’s locations were within ±3 Mbps of the lincRNAs locus (preference assigned to the closest neighbors)*.

*^a^DEG targets potentially relevant in terms of functional aspects, namely RGS22, RGS18 (regulators of G-protein signaling), and STIM2 (stromal interaction molecule 2). Context-rich evidences are richer in functional information compared to only annotated evidences. The annotations reported here are cross-referenced in Table S9 in Supplementary Material (comparatively between species, Ensembl releases, and Ensembl vs. NCBI)*.

In particular, STIM2 plays an important role in ischemia-induced neuronal damage, and its absence in knockout mice was shown to interrupt blood flow in the brain, thus decreasing the neuronal damage caused by ischemia (2). The neuroprotective influence of STIM2-deficiency following an ischemic incidence suggests that the inhibitors of the STIM2 function might have potential therapeutic effect as neuroprotective agents to treat ischemic injury and other neurodegenerative disorders showing altered Ca^2+^ homeostasis. This basically suggests the role of STIM2 in hippocampus-dependent spatial memory, synaptic transmission, and plasticity. Since NP is likely a result of long-term plastic changes along somatosensory pathways, we hypothesize that STIM2 might play a role in NP-related synaptic transmission.

In addition to STIM2, we also detected a few genes from the family of RGS (regulators of G-protein signaling), namely, RGS18 and RGS22, as putative targets for lincRNAs. RGS proteins share a conserved domain of ~120 amino acids and are responsible for accelerating GTPase activity on the G-protein alpha subunit. They also affect physiological regulation of G-protein-mediated cell signaling in many tissues and organs and have been considered as potential drug targets in various nervous system diseases (37, 38). A family of RGS genes expressed in spinal cord may be involved in the regulation of GPCR signaling and adaptive changes of the nervous system, accompanying insensitivity to morphine observed in NP models (39). Based on the expression of RGS proteins, novel targets for small molecule inhibitors might provide specific treatment for various pathophysiological conditions (40).

For instance, RGS18 acts as a negative regulator of the osteoclastogenesis (41) and also plays an essential role in the regulation of megakaryocyte differentiation and chemotaxis (42). Finally, RGS proteins accelerate the deactivation of G proteins to reduce the GPCS (G-protein-coupled receptors) signaling, while few have effector function and thus transmit signals. The range of functions of RGS proteins along with their dynamically regulated distribution in brain makes them putative targets for therapeutic use (43, 44). While there are no current studies that show the relevance of RGS18 and RGS22 to NP, other components of RGS proteins have been shown to contribute to NP etiology (45, 46).

### Annotation Pseudogenes and the Parent Protein Coding

**Figure 3:**
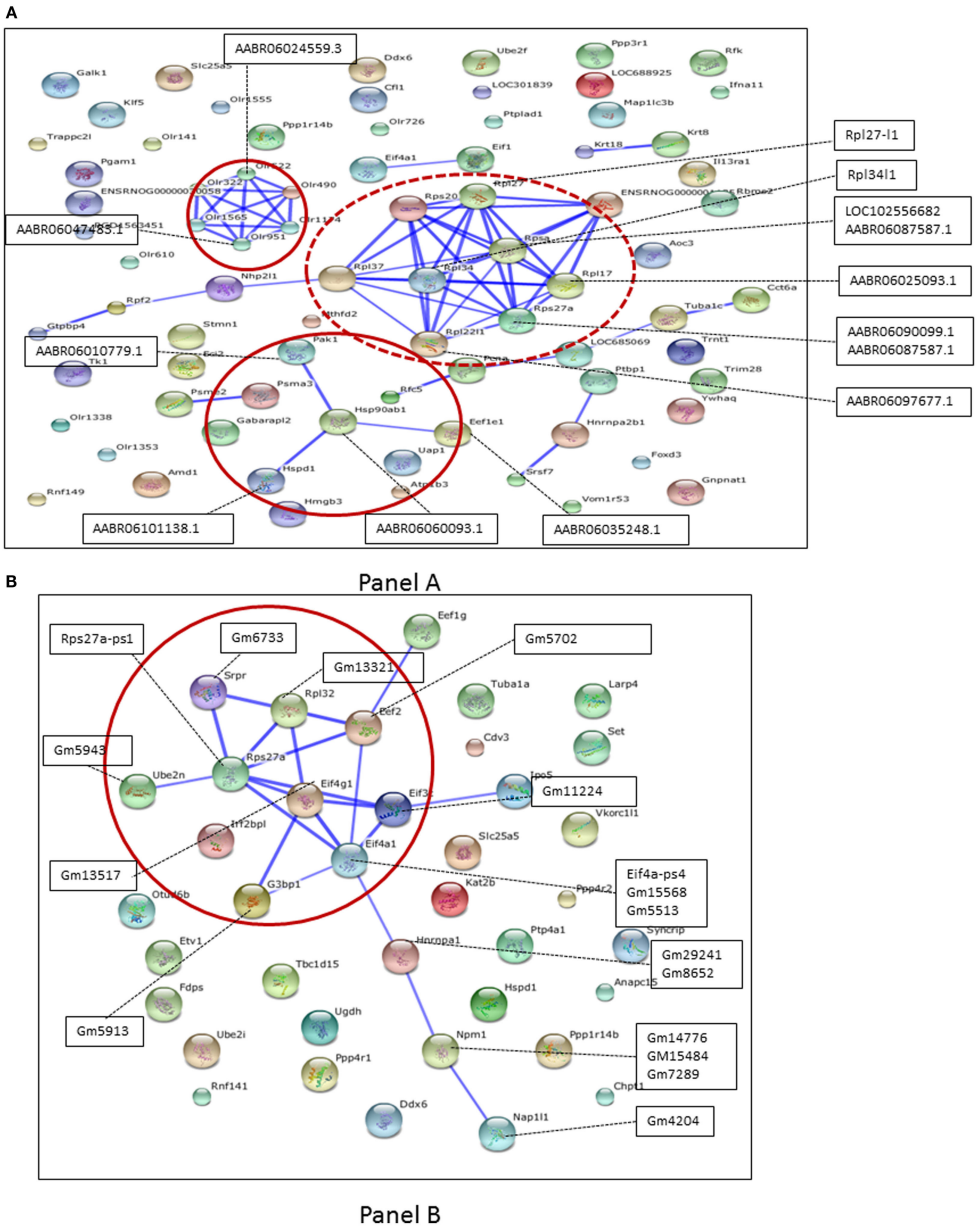
**(A)** Protein–protein interactions for pseudogene targets (black box) obtained using rat reference at confidence level 0.7. Red circle are the networks studied. Dotted lines indicate association between pseudogene and parental genes (the former appear superimposed, being not annotated in STRING). **(B)** Protein–protein interactions for pseudogene targets (black boxes) obtained using mouse reference at confidence level 0.7. Red circle are the networks studied. Dotted lines indicate association between pseudogene and parental genes (the former appear superimposed, being not annotated in STRING).

The contextual analysis of all the pseudogenes detected in both the neuronal tissues was performed. These pseudogenes sequences were mapped to the protein-coding genes to identify their associated parental genes, keeping only the top unique hits for downstream analysis. We further found the differentially expressed genes in either or both of the tissues. The detected pseudogenes and their corresponding parental protein-coding genes were listed in DRG (Table S2 in Supplementary Material) and in SN (Table S3 in Supplementary Material), both at the four different time points. These lists of entities were then used to draw STRING networks (Figure 3A), in an attempt to find the protein–protein interactions involved with pseudogene targets. The rat reference was used as a knowledge base, and a quite stringent confidence level of 0.7 was chosen to control for false positives. The interactions were generated by a database of known and predicted protein–protein interactions, following the STRING structure.

**Figure 4:**
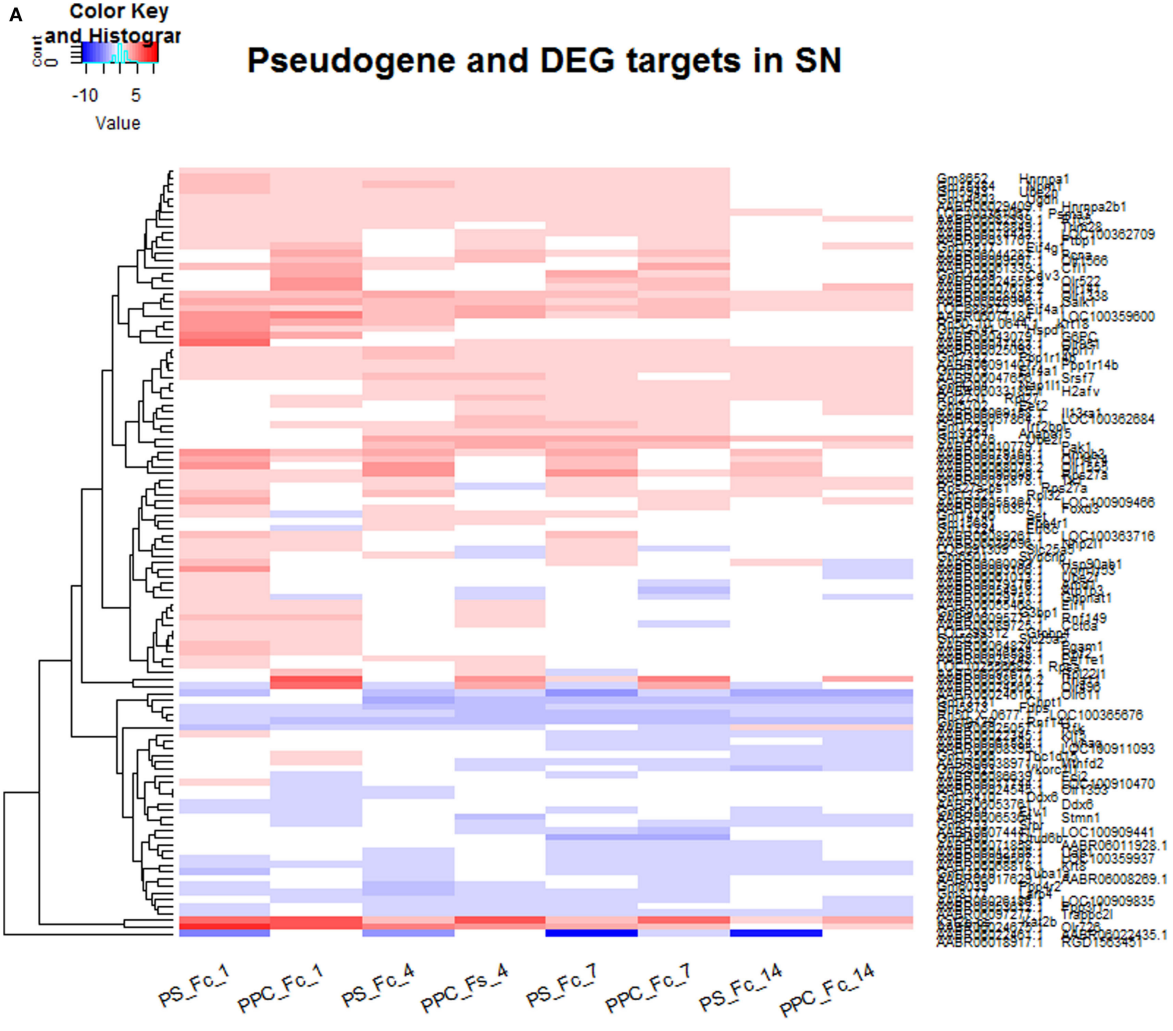

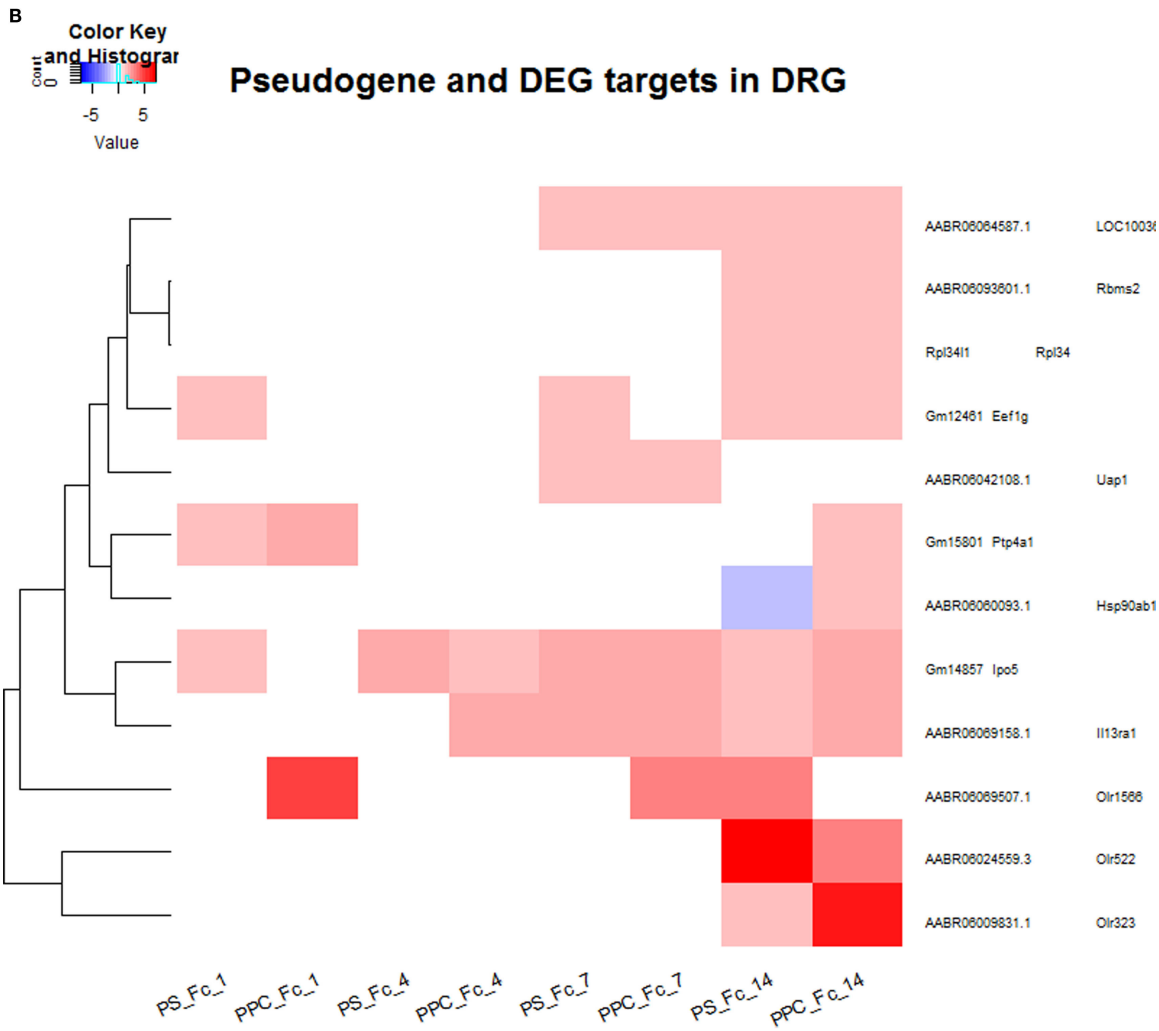
**(A)** Expression levels for pseudogenes and the corresponding parental protein-coding targets that are differentially expressed in SN at four different time points. **(B)** Expression levels for pseudogenes and the corresponding parental protein-coding targets that are differentially expressed in DRG at four different time points.

Figure 3B shows the protein–protein interactions for pseudogene targets (black boxes) obtained using mouse as the reference. Dotted lines indicate associations between pseudogene and protein-coding targets, which have been detected, but for which there is no interaction. In addition, we generated the protein–protein interactions for pseudogene targets obtained using mouse and rat references (at the same confidence level of 0.7, as before) for DRG (Figure S1 in Supplementary Material) and SN (Figure S2 in Supplementary Material). The parental protein-coding targets were further manually curated to match possible transcription factors, kinases, or receptors (shown in Table S1 in Supplementary Material). Protein-coding targets were scrutinized to confirm their role in various neurodegenerative processes. Table 3 reports network-driven identifications of parental genes appearing as paths in Figures 3A,B. Heatmaps were generated for pseudogenes and their DEG targets (Figures 4A,B, for SN and DRG, respectively).

**Table 3:** **Network-driven identifications of parental genes in SN and DRG**.

Rat parental genes	Annotations: relevant gene for which studies are available, with reference model and disease
(DRG) network path: Pak1-Hsp90ab1-Eef1e1-Hspd1	Hspd1 (47) *neurodegeneration* (mouse)
	Hsp90 (48) *neurofibromatosis* (cell lines and human primary schwannoma and meningioma cultures *in vitro*)

**Mouse parental genes**	**Annotations and references**

(SN) network path: Hnrnpa1-Npm1-Nap1l1	Hnrnpa1 (49) *neurodegeneration* (patients)
	Npm1 [(50) – rev.] *neurodegeneration*
	Nap1l1 (51) *neurogenesis* (mouse)
(SN) Network path: Srpr-Ube2n-Rps27a-Rpl32-Eef2-Eef1g-Eif4g1-Eif3c-Eif4a1-G3bp1	Ube2n (52) *neuroblastoma* (cell lines)
	Eef2 (53) *neurogenesis* (*Aplysia californica*)
	Eef1g (54) *neurogenesis* (*in vitro* model of TT2F mouse embryonic stem cells)
	G3BP1 (55) *neurodegeneration* [G3bp1-knockout (KO) mice]

*Network paths are visible in Figures 3 and 4. A path between two nodes in the network is defined as a sequence of adjacent nodes to be traversed in order to go from one of the two nodes to the other*.

Overall, we found that the expression of pseudogenes and their respective targets are more prominent 14 days after injury in DRG, whereas in SN, they are highly expressed at 1, 4, and 7 days after injury. Tables S4 and S5 in Supplementary Material contain the list of pseudogenes and their corresponding parental protein-coding genes in DRG and SN that are differentially expressed in DRG and SN at four different time points, respectively. We found about 12 DEGs in DRG and 134 DEGs in SN to be considered as potential targets for pseudogenes. In particular, we identified a number of olfactory receptors as targets of pseudogenes, which connect according to the network path Olr322-Olr522-Olr490-Olr951-Olr1565-Olr1174 in the rat network and are mostly expressed in SN, with the exception of the path Olr322-Olr522-Olr1565-Olr1174 expressed in DRG as well (Figure S1 in Supplementary Material). In general, these receptors have been reported to be important components for the early diagnosis of neurodegenerative diseases (56–63).

We then identified HSPD1 (Table 3, second row), a heat shock 60-kDA protein1, which encodes a member of the chaperonin family. Studies have shown increased frequency of the pathogenic variant of the HSPD1 single-nucleotide variant in a subgroup of sudden infant death syndrome (SIDS) patients (64). Yet, another study showed that the mutations in HSPD1, the gene encoding Hsp60, are related to two human inherited diseases of the nervous system, spastic paraplegia and MitCHAP60 disease. The study shows the significance of Hsp60 chaperone in mitochondrial function and its relation to the formation of the respiratory chain complex in neuronal tissues (47). We also detected HNRNPA1 in mouse network (Table 3, third row). This is a heterogeneous nuclear ribonucleoprotein A1. The levels of HNRNPA1 were found dysregulated in patients with AD, and the associated genotype is likely a risk factor for frontotemporal lobar degeneration (FTLD) among the male populations (49). This network also includes NAP1l1, which plays a significant role in the cortical neuronal differentiation. NAP1 is the nucleosome assembly protein 1, which is normally used for *in vitro* nucleosome assembly reactions under physiological ionic condition (65). Other studies have shown that NAP1 regulates actin nucleation by forming a complex with WAVE regulatory protein and is selectively expressed in the developing cortex (66). The cytoskeletal rearrangements in the cortical plate play an important role in the cortical neuronal differentiation (51). Yet, another study reveals that the depletion of NAP1 could result in reduced branching of motor axons in the neuronal development (67). Thus, spotting out NAP1 is a significant finding in this study, due to its potential involvement in neurodegeneration.

Naturally enough, we cannot claim any direct association between target genes and both physiological and pathological conditions highlighted in the studies reported in Table 3. Nevertheless, we stress the following two points. (a) The proteins assigned to network paths may or may not refer to significance levels under differentiated models and experimental conditions, but those identified here are indeed obtained from mapped DEGs. (b) A network context is ideal to identify hotspots (motifs, paths, etc.) whose relevance comes from superior robustness of interactions compared to simple correlation observed at gene expression level, which in turn makes inference more reliable.

## Discussion

In recent years, the relevance of ncRNAs has exponentially increased, supported in part by technological advances and in part by the need to overcome the current knowledge limitations in genomics. Two major trends have been observed. On the one hand, the computational community has produced an enormous volume of evidences with a focus on new biotype classifications and characterizations examined in the context of large-scale studies. Undoubtedly, ENSEMBL^3^ represents a main source of genome annotation and interpretation currently available. As prediction remains the most important factor determining genome annotation, the way the available evidence is consolidated and curated differentiates ENSEMBL say, from other annotation systems [see an interesting reading on biotype conflicts, Zhu et al. (68)]. This is one of the current limitations in the analyses we have proposed here, as the lack of harmonization between annotations of different DB resources makes part of the evidences disagree or contradict each other. However, in many cases and especially for the vast majority of ncRNAs, predictive evidences may be destined to change, following discoveries and validations. This is in line with highly focused studies delivered by experimentalists with the aim to provide small-scale validated evidences for identifying functions of ncRNAs. Notably, such research domains, computational and experimental, have offered almost no contact points, due to different scopes, approaches, and desired impacts.

**Figure 5:**
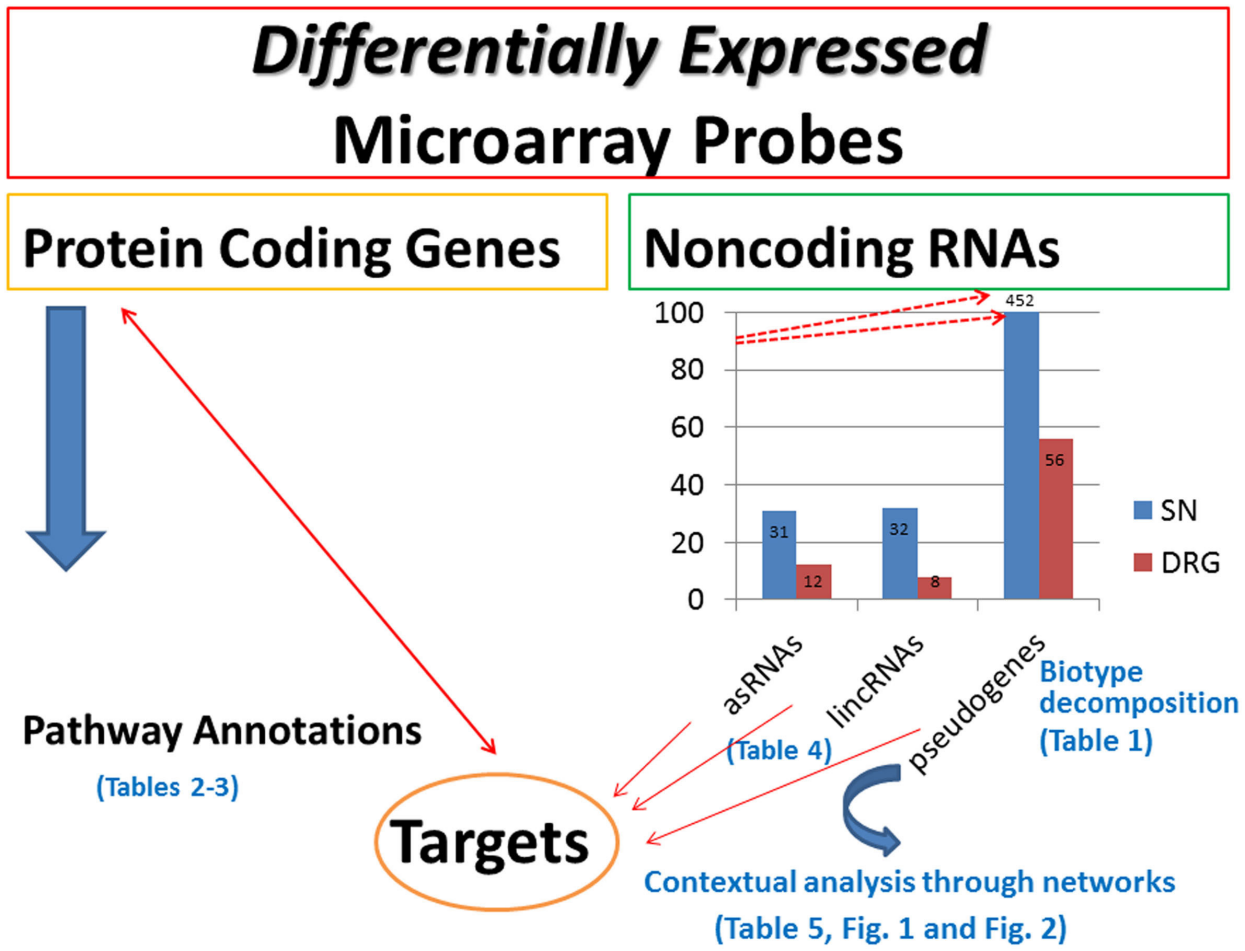
**Methodological pipeline**. The graph has been rescaled to fit the data label (452) for pseudogenes in SN. The upward red arrows pointing to 452 indicate that rescaling has been implemented to fit all the data together. The displayed tables are included in the supplementary material.

We aim to contribute at establishing bridges between the two domains, an effort requiring to computational scientists the implementation of sophisticated tools to parse the current heterogeneity of evidences in support of novel or alternative interpretation especially in relation to the role of ncRNAs. Although microarray targeted to spinal nerve ligations have been performed for real-time evaluation of mRNA transcripts (69, 70), no attempts have been made to study the expression levels of ncRNAs, and specifically for NP studies on spinal cord injury. Here, our idea was to develop a computational pipeline (sketched in Figure 5) to detect and categorize ncRNAs while annotating their putative targets. This way, we emphasize a methodological direction complementing the search and the profiling of protein-coding genes. When these are analyzed with pathway annotations, only a few pathway terms emerged with relevance for our specific problem, while the majority did not convey significant evidence.

In particular, we underline the role of the neurophysiological process_Kappa-type opioid receptor, which is involved in the transmission of nerve impulses and in neurotransmitter release. Such limitations were bypassed by providing further important annotations through protein-coding genes with an assigned role of putative targets of lincRNAs and pseudogenes. Protein interaction networks revealed then very useful to establish connectivity patterns between such target genes. It is important to note that such patterns establish dependence relationships between nodes through links, which reflect an underlying metric. Unlike miRNAs, other ncRNAs are not yet supported by sufficient knowledge to allow complete annotations and systems representation according to networks. For this reason, we focused on building network configurations centered on ncRNA targets, such that inference can be conducted on their direct or indirect interactors.

Previously, it was shown how the differentially expressed genes regulated the nine classes of biological processes, but, here, we did an extensive repurposing of the same microarray data to do pathway annotations for the DEGs. As a disease model, the focus on NP was based on the simple premise that very little is known about the role of ncRNA in such process. It is known that nerve injury causes gene expression changes in some isolated miRNAs and in asRNA in the neuronal tissue. It is also known that these changes could be responsible for the injury-induced alterations of few pain-associated genes, causing neuronal excitability and pain hypersensitivity (22, 71). However, no knowledge is currently available on lncRNAs and their associated protein-coding gene targets. Similar knowledge gap applies also to pseudogenes and their associated parental genes.

We have provided evidence on novel players with a possible role in the regulation of NP development and maintenance, and whose specific functions would surely require further validation steps by experimentalists. As computational scientists, we investigated ncRNA expression profiles and their potential targets by two different approaches. First, we searched for the detected DE lincRNAs in coarsely defined proximal genomic regions to locate potential targets. We found, for instance, three examples of targets – RGS22, RGS18, and STIM2 – for which annotations are quite interesting because of the functional information available for them, hence the attribute context-rich assigned to these examples compared to other examples of candidates for which similar information is unavailable. We do not exclude, of course, that the mechanisms underlying lincRNA functions and their targets might not be centered on proximity. We only claim that evidence appears from targets found at proximal locations, indicating the need to explore further the causes of such influences. While the previous findings were limited to antisense, here we found targets for lincRNAs and pseudogenes, highlighting the fact that the regulation of these targets by ncRNAs needs further exploration in the NP domain.

Second, we analyzed DE pseudogenes and their parental protein-coding targets. We showed that some parental genes can be connected when mapped onto networks, in particular, PINs in our case. Interestingly, we reported various network-driven identifications of these aggregated parental genes linked to studies centered on various neurodegeneration and neurogenesis processes. We also found olfactory receptors representing a densely connected functional module, and further literature curation linked them to various neurodegeneration states like Parkinson, Down syndrome, Huntington diseases, and Alzheimer. This, by no means, indicates that direct association exists with one or more of such diseases, also because of the variety of reference models reported in the studies.

As a methodological note, microarray technology is not comprehensive, and we may expect that by replicating our experimental setting with RNA-Sequence, superior evidences could be found to complement our current results obtained. Although RNA-Sequence is undoubtedly a preferable strategy to detect novel lncRNAs, our goal in the study was limited to the identification of known lncRNAs, and microarray revealed informative for such purpose. We expect that more and novel ncRNAs would emerge from using RNA-Seq. More importantly, we want to emphasize that we have reused the microarray data to identify the significant differentially expressed bioentities and to study their potential relevance for NP. Our results suggest that novel unforeseen findings could be latent in microarray analyses, requiring that biologically meaningful interpretations from such repurposing of microarray probes may represent a strategy to consolidate previously acquired knowledge. Recent reviews stressing such points are supporting our work (72, 73).

In conclusion, given the known premises that lncRNA regulation mechanisms are far from being clarified, and this includes the elucidation of direct/indirect and in case, intermediary mechanisms, it appears that targeting proteins allow to exert their cis or trans effects, but the factors determining, for instance, RNA-protein interaction are not yet well-defined.

Our study brings initial but prominent evidence of the impact exerted by some ncRNAs on NP. Despite the limitations of the approach, such as results telling about putative targets and cross-referencing across studies on different models, an advantage of our methodology is that can be generalized to any model for which even just microarray data have been obtained. To our knowledge, these represent still the major fraction available to computational scientists, and even if the ratio with RNA-Seq-driven studies is destined to revert, we are confident that ncRNA evidences can be retrieved from both experimental sources.

Finally, although most of the recent computational tools have tried to examine the expression and the possible regulation functions of ncRNAs, it is plausible to expect that direct NP implications need future targeted studies aimed to validate the precise functional role of ncRNAs and their potential targets, even if the timeline for such milestone remains highly dependent on both model and type of cells under study, thus hard to predict.

## Author Contributions

HR: performed analyses, computations, coding, and graphics. EC: performed networks and graphics. HR, NT, and EC: designed the methodological approach, wrote the manuscript, read, and approved the final manuscript.

## Conflict of Interest Statement

The authors declare that the research was conducted in the absence of any commercial or financial relationships that could be construed as a potential conflict of interest.

## Footnotes

^1^http://www.ncbi.nlm.nih.gov/geo/query/acc.cgi?acc=GSE30165

^2^http://string-db.org

^3^http://ensemblgenomes.org/

## Supplementary Material

Figure S1 **Protein–protein interactions for pseudogene targets for DRG**.

Figure S2 **Protein–protein interactions for pseudogene targets for SN**.

Table S1 **Parent protein-coding targets classification of transcription factor, kinase, and receptors**.

Table S2 **Pseudogenes and parental protein-coding genes in DRG**.

Table S3 **Pseudogenes and parental protein-coding genes in SN**.

Table S4 **Differentially expressed pseudogenes and parental protein-coding genes in DRG**.

Table S5 **Differentially expressed pseudogenes and parental protein-coding genes in SN**.

Table S6 **Enriched terms and full details for pathway analysis in SN**.

Table S7 **Enriched terms and full details for pathway analysis in DRG**.

Table S8 **Probe sets and genes**.

Table S9 **Cross-references for Table 2 entries**.
