# Supplementary figures and images for: Emerging Putative Associations between Non-Coding RNAs and Protein-Coding Genes in Neuropathic Pain: Added Value from Reusing Microarray Data
Source: Front Neurol. 2016 Oct 18;7:168. doi: 10.3389/fneur.2016.00168 (PMC5067702; doi:10.3389/fneur.2016.00168)

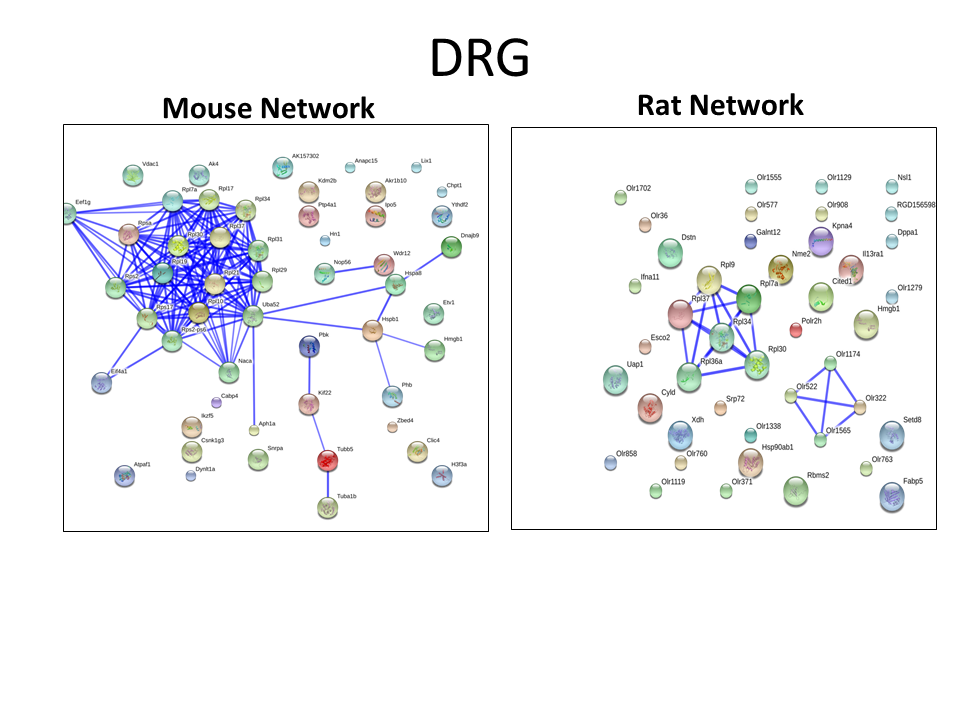

Supplement: Figure S1 — Protein–protein interactions for pseudogene targets for DRG. [file Image_1.tif]

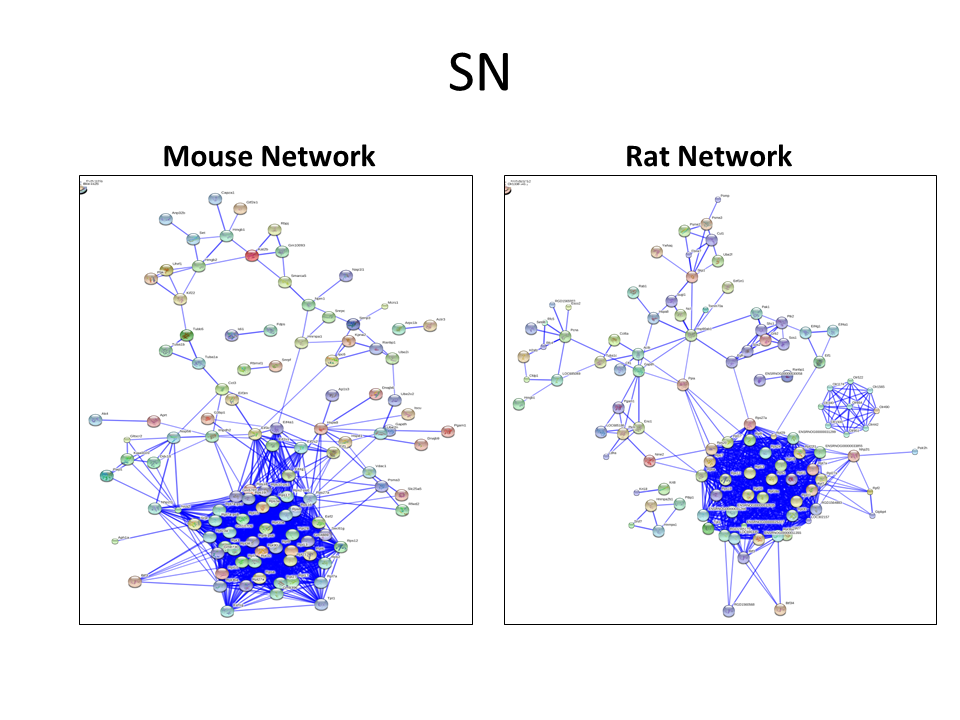

Supplement: Figure S2 — Protein–protein interactions for pseudogene targets for SN. [file Image_2.tif]
