# Supplementary material for: Emerging Putative Associations between Non-Coding RNAs and Protein-Coding Genes in Neuropathic Pain: Added Value from Reusing Microarray Data
Source: Front Neurol. 2016 Oct 18;7:168. doi: 10.3389/fneur.2016.00168 (PMC5067702; doi:10.3389/fneur.2016.00168)
Supplement: Table S1 — Parent protein-coding targets classification of transcription factor, kinase, and receptors. [file Table_1.pdf]

**Transcription Factor**	**Kinase**	**Receptor**
Dbx1	Brsk2	Il13ra1
Elf3	Cdk5	LOC100359937
Pou3f4	Dapk1	Olr1174
Setd6	Dapk3	Olr1338
Taf9b	Galk1	Olr1353
Trim28	Limk1	Olr141
	Mapk4	Olr1555
	Mapk6	Olr1566
	Pak1	Olr323
	Pdpk1	Olr496
	Pxk	Olr522
	Trim13	Olr611
		Olr726
		Olr962
		Srpr
		Srprb
		Traf6
		Vom1r53
